# Patient-Reported Outcome scale for Bronchiectasis (BE-PRO): development and validation in China

**DOI:** 10.1080/07853890.2026.2732346

**Published:** 2026-10-06

**Authors:** Yang Xie, Peng Zhang, Jiajia Wang, Tao Chen, Jiansheng Li

**Affiliations:** ^a^ Department of Respiratory Diseases, National Medical Center, The First Affiliated Hospital, Henan University of Chinese Medicine, Zhengzhou, China; ^b^ Henan International Joint Laboratory of Evidence-Based Evaluation for Respiratory Diseases, The First Affiliated Hospital, Henan University of Chinese Medicine, Zhengzhou, China; ^c^ Collaborative Innovation Center for Chinese Medicine and Respiratory Diseases Co-Construction by Henan Province and Education Ministry of P.R. China/Henan Key Laboratory of Chinese Medicine for Respiratory Diseases, Henan University of Chinese Medicine, Zhengzhou, China; ^d^ Global Health Trials Unit, Department of Clinical Sciences, Liverpool School of Tropical Medicine, Liverpool, UK

**Keywords:** Bronchiectasis, patient-reported outcome measure, quality of life

## Abstract

**Background:**

Patient-reported outcomes (PROs) have become essential endpoints in clinical trials for bronchiectasis. However, no validated PRO instrument currently exists for the Chinese population. This study aimed to develop and validate a disease-specific Patient-Reported Outcome scale for Bronchiectasis (BE-PRO) tailored to Chinese patients.

**Methods:**

A mixed-methods approach was used to develop the BE-PRO. The conceptual framework and initial item pool were developed primarily from semi-structured patient interviews and supplemented by literature and existing-instrument reviews and medical-record analysis. Items were refined through qualitative review by experts and patients. Two field surveys were then conducted, with items selected using classical test theory (CTT), item response theory (IRT), and structural equation modeling (SEM). Finally, a formal validation survey assessed the psychometric properties of the BE-PRO.

**Results:**

The final BE-PRO consists of 16 items organized into five domains: physiology, psychology, environment, society, and treatment satisfaction. The scale demonstrated excellent internal consistency (Cronbach’s *α* = 0.912), split-half reliability (0.856) and satisfactory structural validity (CFI = 0.961; RMSEA = 0.068). Strong correlations with the SF-36 and QOL-B supported criterion validity, while moderate correlations with the BSI and E-FACED supported its clinical relevance. The scale showed high feasibility, with 100% response and completion rates and a mean completion time of 9.07 min.

**Conclusion:**

The 16-item BE-PRO is a reliable, psychometrically validated and feasible instrument for assessing patient-reported outcomes in Chinese patients with bronchiectasis. Its strong psychometric properties support its use in both clinical trials and routine practice.

## Introduction

Bronchiectasis is a chronic respiratory disease characterized by irreversible bronchial dilatation and commonly presents with chronic cough, sputum production, and, in some patients, hemoptysis or dyspnea [1]. Structural lung damage and recurrent exacerbations impose substantial physical, psychological, and socioeconomic burdens [2,3]. Clinical assessment commonly includes symptoms, exacerbation history, sputum microbiology, imaging, and lung function [4]. These measures, however, do not fully capture how the disease and its treatment affect patients’ daily lives. Health-related quality of life (HRQoL) is often significantly impaired in bronchiectasis, worsened by frequent exacerbations, mucus hypersecretion, infection, and psychological comorbidities [5]. Comprehensive assessment should therefore include patient-reported outcomes (PROs) that directly capture symptoms, functioning, and well-being [5,6].

The St. George’s Respiratory Questionnaire (SGRQ), Leicester Cough Questionnaire (LCQ), and Chronic Respiratory Disease Questionnaire (CRDQ) are used in bronchiectasis but were developed for broader respiratory diseases or individual symptoms [7–9]. Chinese versions of the Quality of Life Questionnaire–Bronchiectasis (QOL-B) and Bronchiectasis Health Questionnaire (BHQ) have demonstrated satisfactory measurement properties [10,11]. However, the 37-item QOL-B provides a comprehensive assessment across eight domains but may be less practical for rapid evaluation in routine care [12], whereas the 10-item BHQ offers a brief global assessment but limited domain-specific information [11,13]. Both also retain content developed outside mainland China; validation of their translated versions therefore does not establish comprehensive coverage of concepts important to Chinese patients [14]. A brief instrument combining overall and domain-specific assessment, including environmental influences and treatment satisfaction, is therefore still needed.

Accordingly, this study aimed to develop and validate the Bronchiectasis Patient-Reported Outcome scale (BE-PRO), a multidimensional instrument designed for Chinese patients to provide both overall and domain-specific assessment.

## Methods

The BE-PRO was established through a two-phase process: the development and validation phases. The development flowchart is presented in Figure 1. This study was conducted in accordance with the Declaration of Helsinki (https://www.wma.net/policies-post/wma-declaration-of-helsinki/), with ethical approval from the First Affiliated Hospital of Henan University of Chinese Medicine (No. 2019HL-146-01).

**Figure 1.**
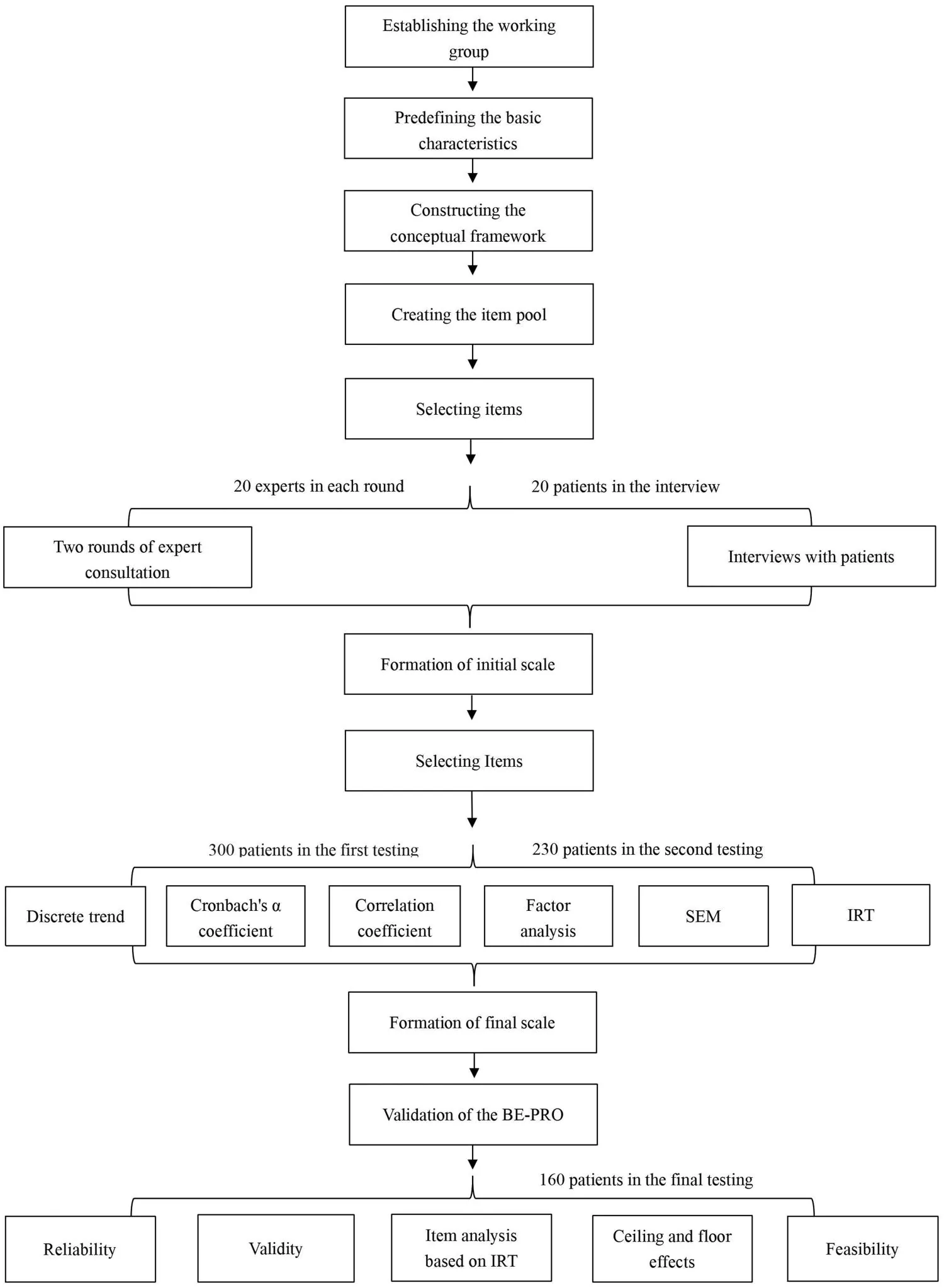
Flowchart of the scale developmental process. BE-PRO, Patient-Reported Outcome scale for Bronchiectasis; IRT, item response theory.

### Study participants

Study participants were recruited from six grade III hospitals across China between June 2021 and November 2021. These included the First Affiliated Hospital of Henan University of Chinese Medicine (Zhengzhou, China), Henan Provincial Hospital of Traditional Chinese Medicine (Zhengzhou China), the Third Affiliated Hospital of Henan University of Chinese Medicine (Zhengzhou, China), Anhui Provincial Hospital of Traditional Chinese Medicine (Hefei, China), Hebei Provincial Hospital of Traditional Chinese Medicine (Shijiazhuang, China), and Liaoning Provincial Hospital of Traditional Chinese Medicine (Shenyang, China).

Eligible participants met the following criteria: (i) clinical diagnosis of bronchiectasis per established criteria [15], (ii) age ≥18 years, (iii) fluency in Chinese, and (iv) signed informed consent. Exclusion criteria included: (a) medical or psychological conditions that could interfere with participation (e.g. pregnancy, critical illness, severe depression/anxiety); and (b) mental illness, impaired consciousness, or significant sensory or communication deficits.

The target sample size followed instrument development guidelines, set at 5–10 times the number of survey items to ensure robust psychometric evaluation [16].

### Development of the BE-PRO

#### Establishing the working group

A study team was formed, consisting of an expert committee and three working groups: Conceptual Framework, Item Management, and the Data Management. Each had distinct roles: The Conceptual Framework Group developed the theoretical model and defined constructs; the Item Management Group handled item generation, revision, and selection; The Data Management Group managed data processing and analysis; The expert committee provided multidisciplinary oversight, with expertise in pulmonology, psychometrics, and statistics.

#### Predefining the basic characteristics

The instrument’s core attributes were predefined: (i) designed for bronchiectasis patients; (ii) disease-specific; (iii) assesses the health status and disease impact; (iv) fewer than 20 items to reduce respondent burden; (v) items organized into distinct conceptual domains; (vi) 5-point Likert scoring (0–4), total score as sum of items; (vii) recall period: ‘past 2 weeks’; (viii) self-report format; (ix) completion time under 15 min in clinical or research settings.

#### Conceptual framework and item pool

The initial item pool was developed primarily through individual, face-to-face, semi-structured interviews with 27 adults with bronchiectasis recruited by convenience sampling. The interview guide covered symptoms, physical functioning, emotional and social impacts, environmental triggers, treatment experiences and other patient-prioritized concerns (Supplementary Table S1). Two researchers independently conducted inductive thematic analysis of contemporaneous interview records, coding relevant statements and grouping them into concepts; disagreements were resolved through discussion. Concepts identified in each interview were compared with the cumulative coding framework, and recruitment ceased when further interviews yielded no new relevant concepts. Patients’ wording was retained where possible, while synonymous expressions were merged and colloquial language standardized without altering the intended meaning. Illustrative mappings of interview-derived concerns to concepts and candidate items are provided in Supplementary Table S2. Interview-derived concepts were supplemented by reviews of the literature and existing health-related quality-of-life instruments and by medical-record review. Overlapping concepts were consolidated and item wording harmonized.

Items were grouped into aspects and domains according to conceptual similarity. A multidisciplinary expert committee reviewed conceptual coverage, domain definitions and item–domain assignments, resolving disagreements by consensus to finalize the item pool and conceptual framework.

#### Qualitative reviews of items to establish the initial scale

The item pool underwent a structured qualitative evaluation involving both clinical experts and patients. Twenty clinical experts independently assessed each item *via* an online questionnaire for relevance, clarity, wording, domain fit, and response format. Expert feedback was analyzed quantitatively, including importance scores, means, coefficients of variation, experts’ positive coefficient, experts’ authority coefficient, experts’ coordination coefficient, and chisquare test. Based on this input, items were refined through panel discussions, involving revision, consolidation, addition, or deletion as appropriate.

Subsequently, cognitive interviews with 20 bronchiectasis patients assessed comprehensibility, relevance, and feasibility. Patient feedback guided further adjustments to wording and structure. This iterative dual-source review produced the preliminary scale.

#### Field testing to establish the final scale

Two consecutive field surveys were conducted. participants independently completed the scale after a standardized introduction. Family assistance was allowed for those with visual or writing difficulties. The final scale was derived through a multi-method framework integrating classical test theory (CTT), item response theory (IRT), and structural equation modeling (SEM).

CTT was applied in both surveys to evaluate item variability, internal consistency, item-domain relationships, and factor analysis. Variability was assessed by standard deviation (SD >0.8). Internal consistency was examined using Cronbach’s *α*, where items increasing *α* upon removal were considered for deletion. Items were required to correlate >0.5 with their domain and the total score, and more strongly with their own domain than others. Exploratory factor analysis (EFA) with orthogonal rotation was conducted, and items with factor loadings <0.4 were excluded [17].

IRT was used in the first survey to estimate discrimination (a) and difficulty (b) parameters. Items were retained if they met: (I) discrimination parameters between 0.3 and 3.0; (II) monotonically increasing difficulty parameters (b1–b4) within the range of −4.0 to 4.0; and (III) mean information >16/n, where n is the total number of items [18].

SEM was applied in the second survey to evaluate items within a multidimensional psychometric framework. The criteria included: (I) retaining items with standardized factor loadings > 0.6 and *p* < 0.05; (II) evaluating model fit using χ^2^/df (1–3), Goodness-of-Fit Index (GFI), Adjusted Goodness-of-Fit Index (AGFI), Comparative Fit Index (CFI), and Normed Fit Index (NFI) > 0.8, and Root Mean Square Error of Approximation (RMSEA) <0.08; (III) confirming convergent validity with composite reliability >0.6 and average variance extracted > 0.5 [19,20]. Items recommended for removal by at least one of the methods (CTT, IRT, or SEM) were excluded from the final scale.

### Validation of the BE-PRO

A subsequent field survey was conducted in patients with bronchiectasis using the finalized scale to evaluate reliability, validity, and feasibility.

#### Reliability

Internal consistency was assessed with Cronbach’s *α* coefficient, and split-half reliability with the Spearman–Brown formula. Both coefficients were acceptable if > 0.70 [21].

#### Validity

Validity was assessed across content, structural, criterion, and discriminant domains. Content validity was evaluated *via* item-domain correlations (≥0.40) [21]. Structural validity used confirmatory factor analysis, with good fit indicated by CFI and NNFI near 0.90 and RMSEA close to 0.08 [20]. Criterion validity was examined *via* Pearson correlations between BE-PRO and SF-36/QOL-B scores (threshold >0.40) [17]. Discriminant validity employed independent-sample *t*-tests comparing high- and low-score groups (top/bottom 27%). To facilitate the clinical interpretation of BE-PRO scores, associations with the Bronchiectasis Severity Index (BSI), Exacerbation-FACED (E-FACED) score, radiological extent, and spirometric indices were examined. Spirometric indices included forced expiratory volume in 1 s (FEV_1_), FEV_1_ percentage predicted, and the FEV_1_/forced vital capacity (FVC) ratio. BSI scores were categorized as low (0–4), intermediate (5–8), or high (≥9), and BE-PRO total scores were compared across these categories.

#### Item analyses based on IRT

The same IRT methods used during item selection were applied in this phase. After confirming unidimensionality through exploratory factor analysis (EFA), discrimination (a) and difficulty (b) parameters were estimated, and item characteristic curves (ICCs) were reviewed. Evaluation criteria for a and b remained consistent with the item selection phase. An optimal ICC showed monotonic first and fifth curves, with the second through fourth curves approximating a normal distribution [18].

#### Ceiling and floor effects

Each item’s response distribution was analyzed for ceiling or floor effects. If over 15% of responses were at the highest or lowest option, a ceiling or floor effect was considered present.

#### Feasibility

Feasibility was assessed using response rate, completion rate, and average completion time. Acceptable thresholds were response/completion rates >85% and average time <20 min [22].

### Statistical analysis

IRT analyses used MULTILOG 7.03; SEM used Amos 26.0 and LISREL 8.80; other analyses used SPSS 26.0. Continuous data are expressed as mean ± standard deviation (SD), categorical data as percentages. Questionnaires with ≥20% missing data were excluded. Missing item-level data were imputed using the item mean.

## Results

### Conceptual framework and item pool

After integration, removal of overlapping content, and wording refinement, the initial item pool comprised 150 items. Of these, 130 were derived from patient interviews, whereas 12 and 8 additional items were identified through literature/instrument review and medical-record review, respectively (Supplementary Table S3). Categorization of the items by conceptual similarity, followed by expert committee review, resulted in a conceptual framework comprising five domains and 20 aspects (Supplementary Table S4): Physiology (eight aspects; 78 items), Psychology (two aspects; 41 items), Environment (four aspects; 4 items), Society (three aspects; 21 items), and Treatment Satisfaction (three aspects; 6 items).

### Establishment of the initial scale

Both consultation rounds were completed by all 20 experts. Expert review and cognitive interviews with 20 patients reduced the 150-item pool to a 60-item initial BE-PRO covering five domains. Patient feedback informed item consolidation and wording refinement. Consultation statistics and item revisions are reported in the Supplementary Results, and the 60-item scale is presented in Supplementary Table S5.

### Establishment of the final scale

Of the 300 questionnaires distributed in the first field survey, 295 were included in the analysis. Evaluation using classical test theory and item response theory reduced the initial scale from 60 to 27 items. In the second survey, 223 of 230 questionnaires were analyzed; further evaluation using classical test theory and structural equation modelling, followed by expert adjudication, yielded the final 16-item BE-PRO. It comprised Physiology (eight items), Psychology (two items), Environment (two items), Society (two items), and Treatment Satisfaction (two items). The complete instrument is provided in Supplementary Materials, and item-level reduction results are reported in Supplementary Tables S6 and S7.

### Validation of the BE-PRO

The final validation included 160 patients with bronchiectasis (62 men and 98 women; mean age 53.4 years). Demographic characteristics are shown in Table 1.

**Table 1.** Details of the patient characteristics.

Variables	Category	First survey for item selection (*n* = 295)	Second survey for item selection (*n* = 223)	final survey for validation (*n* = 160)
Age		66.2 ± 8.7	54.8 ± 13.6	53.4 ± 13.5
Gender	Male	104 (35.2%)	80 (35.9%)	62 (38.8%)
	Female	191 (64.8%)	143 (64.1%)	98 (61.3%)
Marital status	Unmarried	3 (1.0%)	14 (6.3%)	11 (6.9%)
	Married	287 (97.3%)	202 (90.6%)	142 (88.8%)
	Divorced	3 (1.0%)	5 (2.2%)	5 (3.1%)
	Widowed	2 (0.7%)	2 (0.9%)	2 (1.3%)
Educational level	Elementary school and below	129 (43.7%)	63 (28.3%)	42 (26.2%)
	Junior high school	58 (19.7%)	58 (26.0%)	36 (22.5%)
	High school	53 (18.0%)	44 (19.7%)	31 (19.4%)
	College and above	55 (18.6%)	58 (26.0%)	51 (31.9%)
Occupation	Farmer	58 (19.7%)	47 (21.1%)	34 (21.3%)
	Worker	162 (54.9%)	41 (18.4%)	26 (16.3%)
	Professional skill Worker	7 (2.4%)	12 (5.4%)	13 (8.1%)
	Staff	32 (10.8%)	20 (9.0%)	13 (8.1%)
	Manager	20 (6.8%)	11 (4.9%)	7 (4.4%)
	Retired personnel	10 (3.4%)	70 (31.4%)	49 (30.6%)
	Others	6 (2.0%)	22 (9.8%)	18 (11.2%)
Smoking status	Yes	62 (21.0%)	36 (16.1%)	24 (15.0%)
	No	233 (79.0%)	187 (83.9%)	136 (85.0%)
Alcohol consumption	Yes	67 (22.7%)	21 (9.4%)	16 (10.0%)
	No	228 (77.3%)	202 (90.6%)	144 (90.0%)
Clinical characteristics				
BSI category	Low (0–4)	—	—	6 (3.8%)
	Intermediate (5–8)	—	—	104 (31.3%)
	High (≥9)	—	—	50 (65.0%)
Radiological extent	One lobe	—	—	61 (38.1%)
	Two lobes	—	—	46 (28.8%)
	>2 lobes	—	—	53 (33.1%)
Airway microbial colonization	Present	—	—	35 (21.9%)
	Absent	—	—	103 (64.4%)
	Not assessed/unknown	—	—	22 (13.8%)

BSI, Bronchiectasis Severity Index. *Note:* Data are presented as mean ± standard deviation or n (%). Clinical characteristics were available only for the final validation survey. Airway microbial colonization was classified as present if *Pseudomonas aeruginosa* or another microorganism was recorded, absent if both colonization variables were negative, and not assessed/unknown otherwise. Percentages may not total 100 because of rounding.—, data not available.

#### Reliability

The Cronbach’s *α* coefficient for the entire scale was 0.912, with domain-specific values of 0.853 for the physiology domain, 0.926 for the psychology domain, 0.821 for the environment domain, 0.773 for the social domain, and 0.809 for the satisfaction domain. Additionally, the split-half reliability was assessed, yielding a Spearman-Brown coefficient of 0.856 and a Guttman split-half coefficient of 0.853.

#### Content validity

For physiology (*n* = 8), The correlation coefficients between individual item scores and their respective domain ranged 0.703–0.944; for psychology (*n* = 2), 0.894–0.917; for environment (*n* = 2), 0.759–0.923; for society (*n* = 2), 0.892–0.916; and for satisfaction (*n* = 2), 0.887–0.912. Each domain score also correlated substantially with the total scale score (0.586–0.879). Tables 2 and 3 presents the item-domain correlations.

**Table 2.** Correlation coefficient between item scores and domain scores of the BE-PRO.

Domain	Item	Pearson correlation two-sided
Physiological domain	Item 1	0.786*
	Item 2	0.773*
	Item 3	0.750*
	Item 4	0.703*
	Item 5	0.770*
	Item 6	0.776*
	Item 7	0.939*
	Item 8	0.944*
Psychological domain	Item 9	0.917*
	Item 10	0.894*
Environmental domain	Item 11	0.923*
	Item 12	0.759*
Social domain	Item 13	0.916*
	Item 14	0.892*
satisfaction domain	Item 15	0.887*
	Item 16	0.912*

BE-PRO, Patient-Reported Outcome scale for Bronchiectasis. *Note:* *Significant at the *p* < 0.01 level (two-tailed).

**Table 3.** Correlations between the BE-PRO domains and the total scale.

Correlation	Physiological domain	Psychological domain	Environmental domain	Environmental domain	satisfaction domain
Total scale	0.879*	0.765*	0.586*	0.794*	0.794*

BE-PRO, Patient-Reported Outcome scale for Bronchiectasis. *Note:* *Significant at the *p* < 0.01 level (two-tailed).

#### Structural validity

The CFA results supported the hypothesized scale structure. Fit indices were as follows: CFI = 0.961, GFI = 0.892, AGFI = 0.839, NFI = 0.915, and RMSEA = 0.068. Standardized factor loadings for all items ranged from 0.46 to 0.88 in the measurement model (Figure 2).

**Figure 2.**
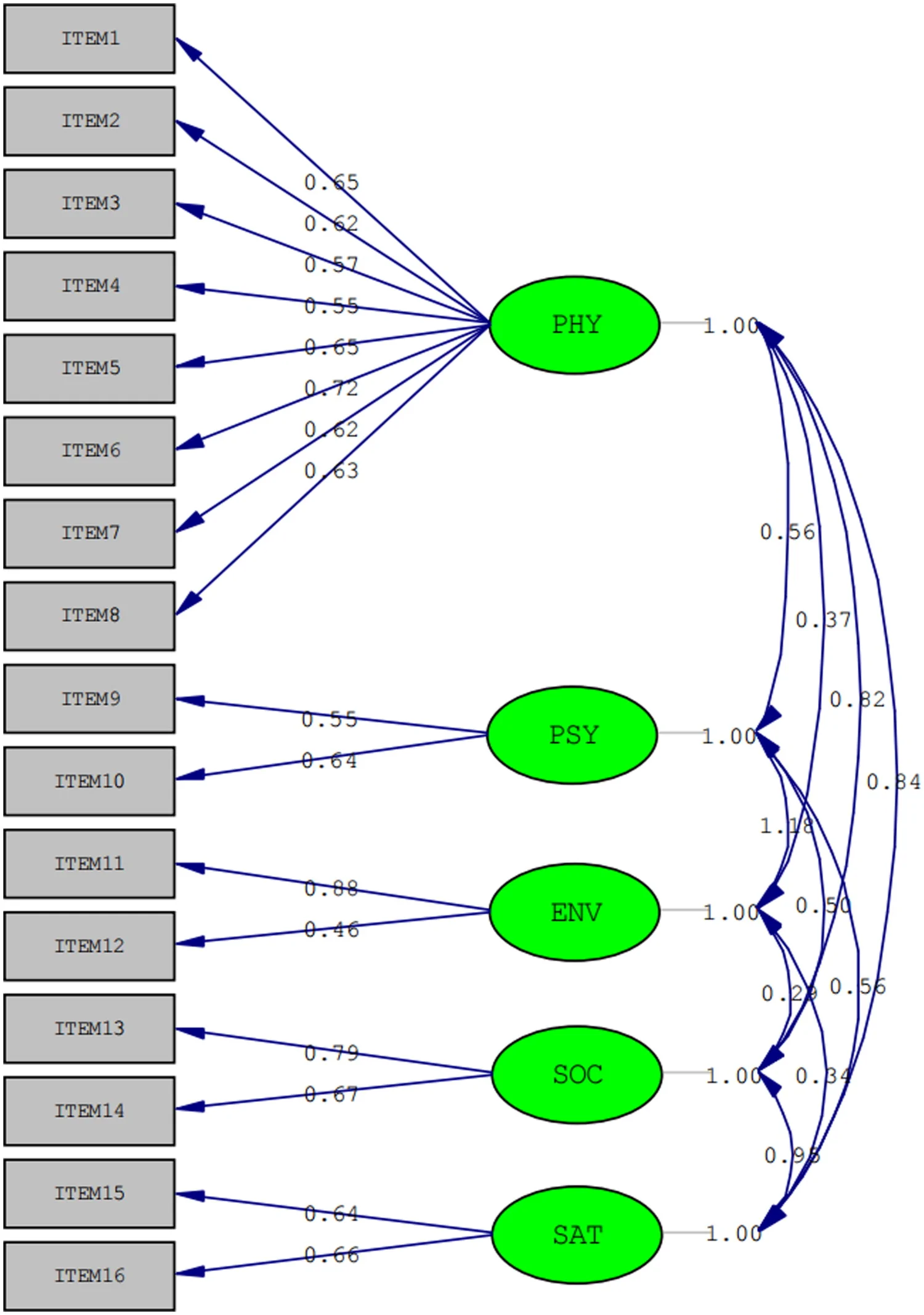
CFA construct frameworks and standard estimates in four domains of the BE-PRO. PHY, Physiology; PSY, Psychology; ENV, Environment; SOC, Society; SAT, Satisfaction

#### Criterion validity

The BE-PRO total scores correlated significantly with established outcome measures. A strong positive correlation was observed with the SF-36 (*r* = 0.773, *p* < 0.01) and with the QOL-B (*r* = 0.800, *p* < 0.01).

#### Discriminant validity

Participants were classified into high- and low-score groups based on the total BE-PRO score distribution. As shown in Supplementary materials (Table S8), independent-sample *t*-tests confirmed statistically significant differences (*p* < 0.01) in each item score between the two groups, supporting the scale’s ability to discriminate across levels of disease impact.

#### Associations with clinical measures

BE-PRO total scores were moderately and positively correlated with the BSI (*ρ* = 0.441, 95% CI 0.307–0.565; *p* < 0.001) and E-FACED score (*ρ* = 0.456, 95% CI 0.313–0.585; *p* < 0.001). Correlations with radiological extent, FEV_1_, FEV_1_ percentage predicted, and FEV_1_/FVC were weak and nonsignificant (all *p* > 0.05; Table 4).

**Table 4.** Associations between BE-PRO total scores and clinical measures.

Clinical measure	*n*	Spearman’s *ρ*	95% CI	*p* Value
BSI	160	0.441	0.307–0.565	<0.001
E-FACED score	160	0.456	0.313–0.585	<0.001
Radiological extent	160	0.089	−0.062–0.238	0.261
FEV₁, L	160	−0.098	−0.250–0.060	0.220
FEV₁, % predicted	160	0.006	−0.150–0.161	0.944
FEV₁/FVC, %	159	−0.033	−0.185–0.117	0.681

BE-PRO, Patient-Reported Outcome scale for Bronchiectasis; BSI, Bronchiectasis Severity Index; CI, confidence interval; E-FACED, Exacerbation-FACED score; FEV_1_, forced expiratory volume in 1 s; FVC, forced vital capacity. *Note:* Correlations were assessed using Spearman’s rank correlation. The 95% CIs were estimated from 10,000 bootstrap samples. Radiological extent was treated as an ordinal variable. All P values were two-sided.

#### IRT-based item analysis

The EFA indicated that the ratio of the first to second eigenvalue was 3.902 (5.822/1.492), exceeding the threshold of 3, supporting scale unidimensionality. Discrimination parameters (a) for all items ranged from 0.81 to 2.70, within the acceptable range of 0.3–3.0. Difficulty parameters (b) spanned −4.55 to 6.88, with 9 of 16 items falling within the desired range of −4.0 to 4.0 (Supplementary materials Table S9). The ICC curves for most items displayed well-distributed five-category response patterns (Figure S1).

#### Ceiling and floor effects

As presented in Table 5, the proportions of respondents selecting the lowest and highest possible scores for each item on the BE-PRO scale ranged from 1.9% to 15.0% and from 0% to 3.1%, respectively. No significant ceiling or floor effects were observed for the scale.

**Table 5.** Proportion of respondents selecting the lowest and highest possible scores for each item on the BE-PRO scale.

Item	Lowest option (%)	Highest option (%)	Item	Lowest option (%)	Highest option (%)
Item 1	4.4	1.3	Item 9	14.6	0.0
Item 2	5.6	1.3	Item 10	3.1	3.1
Item 3	4.4	1.9	Item 11	2.5	3.1
Item 4	14.4	0.0	Item 12	5.6	0.6
Item 5	15.0	0.6	Item 13	8.8	1.3
Item 6	9.4	0.6	Item 14	11.9	0.0
Item 7	12.5	0.6	Item 15	1.9	0.6
Item 8	11.9	0.0	Item 16	4.4	1.9

BE-PRO, Patient-Reported Outcome scale for Bronchiectasis.

#### Feasibility

The BE-PRO demonstrated high feasibility, with both response and completion rates reaching 100%. The mean completion time was 9.07 min.

## Discussion

The BE-PRO was specifically developed and validated for Chinese patients with bronchiectasis, integrating culturally relevant content that distinguish it from existing PRO measures. The final instrument captures respiratory symptoms, emotional consequences, seasonal and weather-related variation, disruption to daily life, perceived burden on others, and treatment-related evaluations. The inclusion of seasonal and weather-related effects is consistent with Chinese health perspectives that emphasize interactions between individuals and their environment [23]. These environmental and treatment-related experiences extend assessment beyond symptoms and physical functioning and strengthen the content relevance of the BE-PRO for Chinese patients.

The conceptual framework and item pool were developed iteratively. A preliminary structure informed by established health-related quality-of-life frameworks – including the Patient-Reported Outcomes Measurement Information System, the World Health Organization Quality of Life assessment and the Chinese Quality of Life Scale – and multidisciplinary expert discussion was subsequently refined using concepts identified through semi-structured patient interviews, supplemented by literature and instrument review and medical-record review [24–26]. After overlapping concepts were consolidated, the 150 candidate items were grouped by conceptual similarity into five domains. The expert committee then reviewed conceptual coverage, domain definitions and item–domain fit. This process linked the framework directly to patient experience while maintaining theoretical and clinical coherence.

Item reduction combined patient and expert review with classical test theory, item response theory and structural equation modelling, thereby reducing reliance on any single selection criterion. The development and evaluation of the BE-PRO broadly align with the Consensus-based Standards for the selection of health Measurement Instruments (COSMIN) [21,27–29]. Specifically, content validity was addressed through patient interviews, expert review and cognitive testing, covering item relevance, comprehensiveness and comprehensibility; structural validity was examined using factor analysis; internal consistency was assessed at the scale and domain levels; and construct validity was evaluated through associations with established PRO measures and clinical severity indicators. Strong correlations with the SF-36 and Quality of Life Questionnaire–Bronchiectasis supported convergent validity, whereas moderate correlations with the Bronchiectasis Severity Index and E-FACED score supported the expected relationship with clinical severity. The absence of significant associations with radiological extent or spirometry suggests that the BE-PRO captures aspects of disease burden not represented by structural or physiological measures. The absence of substantial floor or ceiling effects and a mean completion time of approximately 9 min further supported its feasibility.

The Quality of Life Questionnaire–Bronchiectasis (QOL-B) and Bronchiectasis Health Questionnaire (BHQ) are the principal bronchiectasis-specific PRO instruments. The 37-item QOL-B provides broad coverage of respiratory symptoms, physical and social functioning, emotional well-being and treatment burden, but may impose greater respondent burden [12,30]. The 10-item BHQ is easier to administer, although its brevity necessarily limits content coverage [13]. With 16 items across five domains, the BE-PRO provides an intermediate balance between breadth and brevity. Its principal contribution is not superiority to these instruments, but direct development in Chinese patients and inclusion of environmental and treatment-related experiences that may otherwise be overlooked. Further comparative studies are needed to determine the most appropriate instrument for specific clinical and research contexts.

The BE-PRO could be incorporated into both routine follow-up and clinical research. In routine care, patients could complete the 16-item instrument before or during scheduled visits, allowing total and domain scores to be reviewed longitudinally alongside exacerbation history, sputum microbiology, spirometry, imaging and disease-severity indices [31]. Domain profiles could prompt targeted review of respiratory symptoms, airway-clearance practices, psychosocial difficulties, environmental triggers and treatment burden. Electronic scoring could display score trajectories and flag domains requiring clinical review. Reassessment after a major treatment change could help characterize patient-perceived improvement or deterioration; however, score changes should complement clinical assessment rather than be interpreted in isolation until the minimal clinically important difference has been established. In clinical trials, the BE-PRO could initially be included as a prespecified secondary or exploratory patient-centred endpoint [32]. The total score or relevant domain score should be selected a priori according to the anticipated treatment effect, with assessments conducted at baseline and fixed follow-up points consistent with the 2-week recall period. Endpoint definition, missing-data handling and statistical analysis should be prespecified in accordance with SPIRIT-PRO and CONSORT-PRO recommendations [33,34].

Several limitations should be acknowledged. First, although internal consistency was established, test-retest reliability was not assessed. Because test–retest evaluation requires clinically stable patients, we did not impose strict stability criteria in the present study in order to preserve population representativeness and generalisability. Future studies will assess test–retest reliability and measurement error in a clinically stable cohort using an appropriate retest interval. Second, the cross-sectional design precludes evaluation of responsiveness. Treatment regimens varied among patients with bronchiectasis, limiting the ability to evaluate BE-PRO changes in relation to a defined clinical intervention. Future longitudinal studies using a more standardised treatment approach are warranted to assess responsiveness to clinical improvement or deterioration and to establish the minimal clinically important difference. Third, while rigorous methods were applied, item selection and psychometric evaluation followed widely accepted rather than universally standardized criteria, which may introduce methodological variability across studies.

## Conclusion

The BE-PRO demonstrated favorable reliability, validity, and clinical feasibility, with items showing suitable levels of differentiation and difficulty abilities. Therefore, it may become a reliable and psychometrically validated instrument for bronchiectasis patients in China. However, further studies are needed to optimize its items and verify its measurement properties across more provinces in China.

## Supplementary Material

Revised Supplementary materials.docx

## Data Availability

The datasets generated and analyzed during the current study are available from the corresponding author on reasonable request. The final BE-PRO scale is provided in the supplementary materials accompanying this article.
