# Supplementary material for: Patient-Reported Outcome scale for Bronchiectasis (BE-PRO): development and validation in China
Source: Ann Med. 2026 Oct 6;58(1):2732346. doi: 10.1080/07853890.2026.2732346 (PMC13644423; doi:10.1080/07853890.2026.2732346)
Supplement: Supplementary file 1 [file IANN_A_2732346_SM1877.docx]

**Supplementary Materials**

**Supplementary Qualitative Materials**

**Table S1. Semi-structured concept-elicitation interview guide**

| **Topic** | **Core question** | **Optional probes** | **Purpose for item development** |
| --- | --- | --- | --- |
| Opening and overall experience | Please describe, in your own words, what living with bronchiectasis is like for you. | Main concerns; changes since diagnosis; effects considered most important | Establish the patient’s spontaneous disease experience |
| Symptoms | What symptoms related to bronchiectasis do you experience? | Cough; sputum; hemoptysis; breathlessness; chest discomfort; fatigue; sleep and appetite; frequency, severity, and variability | Identify symptom concepts and patient-preferred expressions |
| Triggers and variability | Are there situations or environmental conditions that make your symptoms better or worse? | Physical activity; seasonal or weather changes; air pollution; irritating odors; temperature changes; confined or poorly ventilated spaces | Identify environmental and activity-related concepts |
| Physical functioning | How does bronchiectasis affect your daily physical activities? | Walking; household tasks; work; self-care; sleep; exercise; need to rest or slow down | Identify activity-limitation and functional-impact concepts |
| Emotional impact | How does bronchiectasis affect your feelings or thoughts? | Worry; fear; low mood; irritability; embarrassment; confidence; concerns about worsening | Identify emotional and cognitive concepts |
| Social and role impact | How does bronchiectasis affect your relationships, social activities, or usual roles? | Family roles; work; leisure; visiting others; social support; feeling burdensome | Identify social-functioning and role-participation concepts |
| Treatment experience | Please describe your experience of treatment for bronchiectasis. | Perceived benefit; treatment burden; side effects; confidence; satisfaction; unmet needs | Identify treatment-experience and satisfaction concepts |
| Prioritization and closing | Which effects of bronchiectasis matter most to you? Is there anything important that we have not discussed? | Most important symptoms or impacts; missing concepts; preferred wording | Confirm content coverage and identify additional concepts |

**Note:** Twenty-seven adults with bronchiectasis were recruited by convenience sampling and interviewed individually in Chinese. Open-ended questions preceded optional probes. Two researchers independently coded the contemporaneous written interview records using inductive thematic analysis and resolved discrepancies through discussion. Interviews continued until no new relevant concepts emerged. Recorded patient expressions were retained where possible when candidate items were formulated.

**Table S2. Illustrative mapping of interview-derived concerns to candidate items**

| **Patient-reported concern (summary)** | **Inductive concept** | **Candidate item** | **Domain** |
| --- | --- | --- | --- |
| 做一些日常活动时，咳嗽会加重。 Cough worsened during routine daily activities. | Activity-related cough | 您感觉您的咳嗽会因适当的活动（扫地、洗菜、散步等）而加重吗？ Does your cough worsen with moderate activities (e.g., sweeping, washing vegetables, or walking)? | Physiology |
| 扫地、走路等活动会让我更气短。 Breathlessness worsened during moderate activity. | Activity-related breathlessness | 您感觉您的气短会因适当的活动（扫地、洗菜、散步等）而加重吗？ Does your breathlessness worsen with moderate activities (e.g., sweeping, washing vegetables, or walking)? | Physiology |
| 咳嗽停不下来时会害怕、心慌。 Fear or panic when coughing could not be stopped. | Cough-related fear or panic | 您咳嗽不止时是否感到恐惧或惊慌？ Do you feel frightened or panicked when your coughing does not stop? | Psychology |
| 阴天、雾霾、刮风或下雨时症状会加重。 Symptoms worsened with changes in weather. | Weather-related symptom aggravation | 天气变化（天阴、雾霾、刮风、下雨）会影响您的症状吗？ Are your symptoms affected by weather changes (e.g., overcast days, smog, wind, or rain)? | Environment |
| 咳嗽会打断说话和接电话。 Coughing interrupted conversations and telephone calls. | Communication restriction | 您会因咳嗽中断谈话或接听电话吗？ Does coughing interrupt your conversations or telephone calls? | Society |
| 最关心治疗后症状有没有改善。 Concern about improvement in symptoms after treatment. | Perceived treatment benefit | 您对症状改善情况是否满意？ Are you satisfied with the improvement in your symptoms? | Treatment Satisfaction |

**Note:** Patient-reported concerns are concise analytic summaries of interview-derived content rather than verbatim quotations. The examples illustrate how interview content was coded into concepts and translated into candidate items while preserving the intended meaning.

The initial item pool integrated concepts elicited in patient interviews and was supplemented by literature/instrument and medical-record reviews. Overlapping concepts were consolidated and wording was standardized. Item numbers are provided for presentation only and do not indicate the order in which concepts were generated.

**Table S3. Initial 150-item pool of the Patient-Reported Outcome scale for Bronchiectasis (BE-PRO) and source attribution**

| **No.** | **Domain** | **Candidate item (Chinese)** | **English translation** | **Primary source** |
| --- | --- | --- | --- | --- |
| 1 | Physiology | 您咳嗽时伴有大便失禁吗？ | Does coughing cause fecal incontinence? | PI |
| 2 | Physiology | 您感觉您的咳嗽会因适当的活动（扫地、洗菜、散步等）而加重吗？ | Does your cough worsen with moderate activities (e.g., sweeping, washing vegetables, or walking)? | PI |
| 3 | Physiology | 您感觉您的咳嗽会因剧烈活动（跑步、搬重物、爬山等）而加重吗？ | Does your cough worsen with strenuous activities (e.g., running, carrying heavy objects, or climbing)? | PI |
| 4 | Physiology | 您感觉您的咳嗽会因异常刺激（刺激性气味、瞬间的气温变化）而加重吗？ | Does your cough worsen with unusual stimuli (e.g., irritating odors or sudden temperature changes)? | PI |
| 5 | Physiology | 您是否咳嗽得打电话讲话困难？ | Does coughing make it difficult for you to speak on the telephone? | PI |
| 6 | Physiology | 咳嗽会让您胸痛或肚子痛吗？ | Does coughing cause chest or abdominal pain? | PI |
| 7 | Physiology | 您感觉您的咯痰会因剧烈活动（跑步、搬重物、爬山等）而加重吗？ | Does your sputum production worsen with strenuous activities (e.g., running, carrying heavy objects, or climbing)? | PI |
| 8 | Physiology | 您感觉您的咯痰会因异常刺激（刺激性气味、瞬间的气温变化）而加重吗？ | Does your sputum production worsen with unusual stimuli (e.g., irritating odors or sudden temperature changes)? | PI |
| 9 | Physiology | 您咯黑黏痰吗？ | Do you produce black, sticky sputum? | MRR |
| 10 | Physiology | 您感觉您的胸闷会因适当的活动（扫地、洗菜、散步等）而加重吗？ | Does your chest tightness worsen with moderate activities (e.g., sweeping, washing vegetables, or walking)? | PI |
| 11 | Physiology | 您感觉您的胸闷会因剧烈活动（跑步、搬重物、爬山等）而加重吗？ | Does your chest tightness worsen with strenuous activities (e.g., running, carrying heavy objects, or climbing)? | PI |
| 12 | Physiology | 您感觉您的胸闷会因异常刺激（刺激性气味、瞬间的气温变化）而加重吗？ | Does your chest tightness worsen with unusual stimuli (e.g., irritating odors or sudden temperature changes)? | PI |
| 13 | Physiology | 您弯腰时会喘不过来气吗？ | Do you become short of breath when bending over? | PI |
| 14 | Physiology | 您感觉您的气喘会因适当的活动（扫地、洗菜、散步等）而加重吗？ | Does your wheezing worsen with moderate activities (e.g., sweeping, washing vegetables, or walking)? | PI |
| 15 | Physiology | 您感觉您的气喘会因剧烈活动（跑步、搬重物、爬山等）而加重吗？ | Does your wheezing worsen with strenuous activities (e.g., running, carrying heavy objects, or climbing)? | PI |
| 16 | Physiology | 您感觉您的气喘会因异常刺激（刺激性气味、瞬间的气温变化）而加重吗？ | Does your wheezing worsen with unusual stimuli (e.g., irritating odors or sudden temperature changes)? | PI |
| 17 | Physiology | 您感觉您的气短会因适当的活动（扫地、洗菜、散步等）而加重吗？ | Does your breathlessness worsen with moderate activities (e.g., sweeping, washing vegetables, or walking)? | PI |
| 18 | Physiology | 您感觉您的气短会因剧烈活动（跑步、搬重物、爬山等）而加重吗？ | Does your breathlessness worsen with strenuous activities (e.g., running, carrying heavy objects, or climbing)? | PI |
| 19 | Physiology | 您感觉您的气短会因异常刺激（刺激性气味、瞬间的气温变化）而加重吗？ | Does your breathlessness worsen with unusual stimuli (e.g., irritating odors or sudden temperature changes)? | PI |
| 20 | Psychology | 体育运动对您来说是否不安全？ | Do you feel that physical exercise is unsafe for you? | LIR |
| 21 | Physiology | 您是否做任何事情都很吃力？ | Does everything you do require considerable effort? | LIR |
| 22 | Physiology | 您是否走得比别人慢，或需要停下来歇歇？ | Do you walk more slowly than other people or need to stop and rest? | LIR |
| 23 | Physiology | 您是否需要限制一些日常活动？ | Do you need to limit any of your usual daily activities? | LIR |
| 24 | Physiology | 您胸部有针刺样痛吗？ | Do you have stabbing chest pain? | MRR |
| 25 | Physiology | 您胸部有隐隐作痛吗？ | Do you have a dull ache in your chest? | MRR |
| 26 | Physiology | 您有胸部胀痛吗？ | Do you have distending chest pain? | MRR |
| 27 | Physiology | 您的胸痛会于夜间加重吗？ | Does your chest pain worsen at night? | PI |
| 28 | Physiology | 您有多关节肿痛吗？ | Do you have swelling and pain in multiple joints? | MRR |
| 29 | Physiology | 您是否头疼？ | Do you have headaches? | PI |
| 30 | Physiology | 您是否眩晕？ | Do you experience dizziness? | PI |
| 31 | Physiology | 您是否浑身疼？ | Do you have pain throughout your body? | PI |
| 32 | Physiology | 您有恶心和（或）呕吐吗？ | Do you experience nausea and/or vomiting? | MRR |
| 33 | Physiology | 您有耳鸣和（或）耳聋吗？ | Do you have tinnitus and/or hearing loss? | MRR |
| 34 | Physiology | 您觉得口渴吗？ | Do you feel thirsty? | PI |
| 35 | Physiology | 您觉得口黏吗？ | Do you have a sticky sensation in your mouth? | PI |
| 36 | Physiology | 您觉得咽痒吗？ | Do you have an itchy throat? | PI |
| 37 | Physiology | 您觉得口中味觉减退吗？ | Has your sense of taste diminished? | MRR |
| 38 | Physiology | 您平时怕冷吗？ | Are you usually sensitive to cold? | PI |
| 39 | Physiology | 您平时怕风吗？ | Are you usually sensitive to wind? | PI |
| 40 | Physiology | 您平时出汗吗？ | Do you usually sweat easily? | PI |
| 41 | Psychology | 您呼吸困难时是否感到害怕或惊恐？ | Do you feel frightened or panicked when you have difficulty breathing? | LIR |
| 42 | Psychology | 您是否觉得您无法控制您的呼吸问题？ | Do you feel unable to control your breathing problems? | PI |
| 43 | Psychology | 您是否觉得您的呼吸问题能好转？ | Do you think your breathing problems can improve? | PI |
| 44 | Psychology | 目前的治疗方法能给您带来信心吗？ | Does your current treatment give you confidence? | PI |
| 45 | Psychology | 您是否对自己的生活感到高兴或满意？ | Are you happy or satisfied with your life? | PI |
| 46 | Psychology | 您对外出活动有没有信心？ | Do you feel confident about going out? | PI |
| 47 | Psychology | 您是否感到害怕，好像预感到有什么可怕的事情要发生？ | Do you feel frightened, as if something terrible is about to happen? | LIR |
| 48 | Psychology | 咳嗽会让您担心有可能得了重病吗？ | Does coughing make you worry that you may have a serious illness? | PI |
| 49 | Psychology | 您是否觉得疲惫或呆滞？ | Do you feel tired or sluggish? | PI |
| 50 | Psychology | 您是否觉得不适或害怕？ | Do you feel uneasy or frightened? | PI |
| 51 | Psychology | 您是否觉得不安或紧张？ | Do you feel restless or tense? | PI |
| 52 | Psychology | 您是否心中充满烦恼？ | Is your mind full of worries? | PI |
| 53 | Psychology | 您是否能够安闲而轻松地坐着？ | Can you sit at ease and feel relaxed? | PI |
| 54 | Psychology | 您是否对自己的仪容（打扮自己）失去了兴趣？ | Have you lost interest in your appearance or grooming? | LIR |
| 55 | Psychology | 您是否有点坐立不安，好像感到非要活动不可？ | Do you feel restless, as if you must keep moving? | LIR |
| 56 | Psychology | 您是否对一些事情都乐观地向前看？ | Are you able to look forward to things with optimism? | PI |
| 57 | Psychology | 您是否会突然出现恐慌感？ | Do you experience sudden feelings of panic? | LIR |
| 58 | Psychology | 您觉得自己的身体比其他人弱吗？ | Do you feel physically weaker than other people? | PI |
| 59 | Psychology | 您是否感到周围有人在谈论您？ | Do you feel that people around you are talking about you? | PI |
| 60 | Psychology | 您担心您的疾病会传染他人吗？ | Are you worried that your condition may be contagious to others? | PI |
| 61 | Psychology | 您有战胜疾病的信心吗？ | Are you confident that you can overcome your condition? | PI |
| 62 | Psychology | 您能集中注意力吗？ | Are you able to concentrate? | PI |
| 63 | Psychology | 您因为您的疾病而感到孤独吗？ | Do you feel alone because of your condition? | PI |
| 64 | Psychology | 您因为您的疾病而感到寂寞吗？ | Do you feel lonely because of your condition? | PI |
| 65 | Psychology | 您因为您的疾病而感到自责吗？ | Do you blame yourself because of your condition? | PI |
| 66 | Society | 您的疾病使您只能完成一部分自己想做的事情吗？ | Does your condition allow you to complete only some of the things you want to do? | LIR |
| 67 | Society | 您的疾病使您在工作、家务或其他日常活动中需要额外的努力吗？ | Does your condition require extra effort during work, household tasks, or other daily activities? | LIR |
| 68 | Society | 咳嗽及呼吸困难是否让您在他人面前感到难堪？ | Do coughing and breathing difficulties make you feel embarrassed in front of others? | LIR |
| 69 | Society | 您的疾病给家里带来经济负担了吗？ | Has your condition placed a financial burden on your family? | PI |
| 70 | Society | 您是否因为疾病不得不放弃自己的某些兴趣爱好？ | Have you had to give up any hobbies because of your condition? | PI |
| 71 | Society | 您是否觉得自己过着正常的生活？ | Do you feel that you are living a normal life? | PI |
| 72 | Society | 您对自己从亲戚朋友得到的支持满意吗？ | Are you satisfied with the support you receive from relatives and friends? | PI |
| 73 | Society | 您的疾病影响您照顾自己吗（洗脸、刷牙、洗头、穿衣、吃饭等）？ | Does your condition affect your ability to care for yourself (e.g., washing, brushing your teeth, dressing, or eating)? | PI |
| 74 | Society | 您有没有经常感到孤独？ | Do you often feel lonely? | PI |
| 75 | Society | 您的疾病影响您做家务吗（整理房间、洗衣、做饭、洗碗）？ | Does your condition affect your ability to perform household tasks (e.g., cleaning, laundry, cooking, or washing dishes)? | PI |
| 76 | Society | 您的疾病影响您照顾家人吗（照顾父母、孩子、爱人）？ | Does your condition affect your ability to care for family members (e.g., parents, children, or spouse)? | PI |
| 77 | Society | 您的疾病影响您参加休闲娱乐活动吗（看报纸、听广播、看电视、下棋、打牌）？ | Does your condition affect your participation in leisure activities (e.g., reading newspapers, listening to the radio, watching television, playing chess, or playing cards)? | PI |
| 78 | Society | 您会觉得咳嗽惹恼了同伴、家人或朋友？ | Do you feel that your cough annoys your companions, family members, or friends? | PI |
| 79 | Physiology | 您咳嗽吗？ | Do you have a cough? | PI |
| 80 | Physiology | 您有干咳吗？ | Do you have a dry cough? | PI |
| 81 | Physiology | 您咳嗽时伴有小便失禁吗？ | Does coughing cause urinary incontinence? | PI |
| 82 | Physiology | 您有咳嗽无力吗？ | Do you have a weak cough? | PI |
| 83 | Physiology | 您是否咳嗽得出汗？ | Does coughing make you sweat? | PI |
| 84 | Physiology | 您咯白黏痰吗？ | Do you produce white, sticky sputum? | PI |
| 85 | Physiology | 您咯黄稠痰吗？ | Do you produce yellow, thick sputum? | PI |
| 86 | Physiology | 您咯痰时有痰中带血吗？ | Is there blood in your sputum? | PI |
| 87 | Physiology | 您有喉中痰鸣吗？ | Do you have phlegm rattling in your throat? | PI |
| 88 | Physiology | 您是否咯血？ | Do you have hemoptysis? | PI |
| 89 | Physiology | 您痰量多吗？ | Do you produce a large amount of sputum? | PI |
| 90 | Physiology | 您的痰容易咯出吗？ | Is it easy for you to expectorate sputum? | PI |
| 91 | Physiology | 您觉得胸闷吗？ | Do you have chest tightness? | PI |
| 92 | Physiology | 您有气喘吗？ | Do you have wheezing? | PI |
| 93 | Physiology | 您说话时会感到喘不过气来吗？ | Do you feel breathless when speaking? | PI |
| 94 | Physiology | 您觉得气短吗？ | Do you feel short of breath? | PI |
| 95 | Physiology | 若赶时间或快走，您是否不得不停下来休息或放慢速度？ | When in a hurry or walking briskly, do you need to stop to rest or slow down? | PI |
| 96 | Physiology | 您是否咳嗽得呼吸引起疼痛？ | Does coughing cause pain when you breathe? | PI |
| 97 | Physiology | 您有胸痛吗？ | Do you have chest pain? | PI |
| 98 | Physiology | 您睡眠情况怎么样？ | How is your sleep? | PI |
| 99 | Physiology | 您胃口怎么样？ | How is your appetite? | PI |
| 100 | Physiology | 您精力怎么样？ | How is your energy level? | PI |
| 101 | Physiology | 您觉得疲劳吗？ | Do you feel fatigued? | PI |
| 102 | Physiology | 您觉得口干吗？ | Do you have dry mouth? | PI |
| 103 | Physiology | 您容易感冒吗？ | Are you prone to catching colds? | PI |
| 104 | Physiology | 您有低热吗？ | Do you have a low-grade fever? | PI |
| 105 | Physiology | 您有腰酸吗？ | Do you have lower-back soreness? | PI |
| 106 | Physiology | 您大小便正常吗？ | Are your bowel and bladder functions normal? | PI |
| 107 | Physiology | 您有口唇、爪甲发绀吗？ | Have you noticed cyanosis of your lips or nails? | PI |
| 108 | Psychology | 咳嗽会让您焦虑不安吗？ | Does coughing make you feel anxious? | PI |
| 109 | Psychology | 咳嗽会让您厌烦甚至痛苦吗？ | Does coughing make you feel annoyed or distressed? | PI |
| 110 | Psychology | 您会因咳嗽而情绪低落吗？ | Does coughing make you feel depressed? | PI |
| 111 | Psychology | 咳嗽会让您觉得尴尬吗？ | Does coughing make you feel embarrassed? | PI |
| 112 | Psychology | 您是否感到沮丧或急躁？ | Do you feel frustrated or irritable because of your condition? | PI |
| 113 | Psychology | 您咳嗽不止时是否感到恐惧或惊慌？ | Do you feel frightened or panicked when your coughing does not stop? | PI |
| 114 | Psychology | 您是否觉得有信心可以治疗疾病？ | Are you confident that your condition can be treated? | PI |
| 115 | Psychology | 您是否对以往感兴趣的事情仍有兴趣？ | Are you still interested in things that previously interested you? | PI |
| 116 | Psychology | 您担心因为您的疾病而被人歧视吗？ | Are you worried about being discriminated against because of your condition? | PI |
| 117 | Psychology | 您担心自己的疾病会逐渐加重吗？ | Are you worried that your condition will gradually worsen? | PI |
| 118 | Psychology | 在对疾病的抗争中，您感到失望吗？ | Do you feel disappointed in your struggle with the condition? | PI |
| 119 | Psychology | 您因为您的疾病而感到自己没有什么价值吗？ | Do you feel worthless because of your condition? | PI |
| 120 | Psychology | 您是否担心健康会恶化？ | Are you worried that your health will deteriorate? | PI |
| 121 | Environment | 季节变化会加重您的症状吗？ | Do seasonal changes worsen your symptoms? | PI |
| 122 | Environment | 天气变化（天阴、雾霾、刮风、下雨）会影响您的症状吗？ | Are your symptoms affected by weather changes (e.g., overcast days, smog, wind, or rain)? | PI |
| 123 | Environment | 密闭或通风不良的环境对您的病情有影响吗？ | Is your condition affected by confined or poorly ventilated environments? | PI |
| 124 | Society | 您的疾病影响您的工作吗？ | Does your condition affect your work? | PI |
| 125 | Society | 您的疾病是否让您的家人、朋友及邻居感到烦恼？ | Does your condition trouble your family, friends, or neighbors? | PI |
| 126 | Society | 您的疾病是否影响您的外出娱乐或消遣？ | Does your condition affect your outdoor leisure or recreational activities? | PI |
| 127 | Society | 您的疾病使您觉得自己是别人的负担吗？ | Does your condition make you feel like a burden to others? | PI |
| 128 | Society | 您的疾病影响您的社交活动吗？（走亲访友） | Does your condition affect your social activities, such as visiting relatives or friends? | PI |
| 129 | Society | 您会因咳嗽中断谈话或接听电话吗？ | Does coughing interrupt your conversations or telephone calls? | PI |
| 130 | Society | 您对自己从家庭得到的支持满意吗？（如精神鼓励、经济支持、体力援助等） | Are you satisfied with the support you receive from your family (e.g., emotional encouragement, financial support, or physical assistance)? | PI |
| 131 | Treatment Satisfaction | 您认为您目前的健康状况如何？ | How would you rate your current health status? | PI |
| 132 | Treatment Satisfaction | 您对当前的治疗效果是否满意？ | Are you satisfied with the current treatment outcome? | PI |
| 133 | Treatment Satisfaction | 您对症状改善情况是否满意？ | Are you satisfied with the improvement in your symptoms? | PI |
| 134 | Treatment Satisfaction | 您对自己日常活动能力满意吗？ | Are you satisfied with your ability to perform daily activities? | PI |
| 135 | Treatment Satisfaction | 您对他人给予的关心、支持是否满意？（家人、朋友、同事、邻居、医护人员等） | Are you satisfied with the care and support you receive from others (e.g., family, friends, colleagues, neighbors, or healthcare professionals)? | PI |
| 136 | Psychology | 目前的治疗方法能够提高您的信心吗？ | Does the current treatment increase your confidence? | PI |
| 137 | Treatment Satisfaction | 您担心药物副作用吗？ | Are you worried about medication side effects? | PI |
| 138 | Physiology | 您有久咳不愈吗？ | Do you have a persistent cough? | PI |
| 139 | Physiology | 您的咳嗽能把您吵醒吗？ | Does your cough wake you from sleep? | PI |
| 140 | Physiology | 您是否咳嗽得声音嘶哑？ | Does coughing make your voice hoarse? | PI |
| 141 | Physiology | 你是否咳嗽得晚上不能睡觉？ | Does coughing prevent you from sleeping at night? | PI |
| 142 | Physiology | 您咳嗽时咳出粘液了吗？ | Do you cough up mucus? | PI |
| 143 | Physiology | 您觉得咽干吗？ | Do you have a dry throat? | PI |
| 144 | Physiology | 您觉得咽痛吗？ | Do you have a sore throat? | PI |
| 145 | Physiology | 您觉得能控制住咳嗽吗？ | Do you feel able to control your cough? | PI |
| 146 | Psychology | 您对将来有信心吗？ | Are you confident about the future? | PI |
| 147 | Physiology | 您的疾病是否会因日常活动而加重？ | Does your condition worsen with daily activities? | PI |
| 148 | Environment | 接触刺激性气味（油漆、油烟、汽车尾气、化工燃料）会加重您的症状吗？ | Does exposure to irritating odors (e.g., paint, cooking fumes, vehicle exhaust, or chemical fuels) worsen your symptoms? | PI |
| 149 | Physiology | 您感觉身体有疼痛吗？ | Do you experience bodily pain? | PI |
| 150 | Society | 您的疾病影响您的日常生活吗？ | Does your condition affect your daily life? | PI |

Abbreviations: BE-PRO, patient-reported outcome scale for bronchiectasis; PI, patient interview; LIR, literature/instrument review; MRR, medical-record review.

**Note:** Primary source denotes the source assigned during item-pool consolidation. Source categories could overlap conceptually, but each item is presented under one primary source. The distribution was PI, n = 130; LIR, n = 12; and MRR, n = 8.

**Supplementary Results**

**Table S4. Conceptual framework of the BE-PRO**

| **Concept** | **Domain** | **Aspects** |
| --- | --- | --- |
| Patient-reported outcome | Physiological domain | primary symptoms, concomitant symptoms and influencing factors, impact on daily life, activity ability, diet, urination & defecation, common cold, sleep |
|  | Psychological  domain | Negative emotions (anxiety, depression, etc.), positive emotions (treatment confidence, positive optimism, etc.) |
|  | Environmental domain | seasonal changes, temperature differences, environmental pollution (air, physical, chemical), external stimuli |
|  | Social domain | social relationships, economic burden, and medical convenience |
|  | Satisfaction domain | satisfaction with disease treatment, satisfaction with disease improvement, and satisfaction with disease understanding |

Abbreviations: BE-PRO, patient-reported outcome scale for bronchiectasis.

**Expert consultation and cognitive review**

All 20 experts completed both consultation rounds. In the first round, the expert authority and coordination coefficients were 0.81 and 0.91, respectively (χ² = 149, P < 0.05). Seventy-eight items were removed; content from 29 of these items was incorporated into retained items, and eight retained items were reworded, leaving 72 items. In the second round, the corresponding coefficients were 0.86 and 0.86 (χ² = 71, P < 0.05). Twelve items were removed, including four whose content was incorporated into retained items, and 19 retained items were reworded. Cognitive interviews with 20 patients further informed item refinement. Cough-related emotional items were consolidated into a symptom-anchored item, examples were added to clarify weather-related triggers, and cyanosis was described in plain language. These procedures yielded the 60-item initial BE-PRO (Table S5).

**Table S5. Initial 60-item BE-PRO following expert consultation and cognitive review**

| **Domain** | **Items** |
| --- | --- |
| Physiological domain | 1. Do you have a cough? |
|  | 2.Do you have a dry cough? |
|  | 3.Does coughing cause urinary incontinence? |
|  | 4.Do you feel weak when coughing? |
|  | 5.Does coughing cause sweating? |
|  | 6.Do you produce sputum? |
|  | 7.Is there blood in your sputum? |
|  | 8.Do you have wheezing in the throat? |
|  | 9.Do you have hemoptysis? |
|  | 10.Do you produce a large amount of sputum? |
|  | 11.Is it easy for you to expectorate sputum? |
|  | 12.Do you have chest tightness? |
|  | 13.Do you have shortness of breath? |
|  | 14.Do you feel breathless when speaking? |
|  | 15.Do you feel short of breath? |
|  | 16.When in a hurry or walking briskly, do you need to stop to rest or slow down? |
|  | 17.Do you have chest pain? |
|  | 18.Does your condition worsen due to daily activities? |
|  | 19.How is your sleep? |
|  | 20.How is your appetite? |
|  | 21.How is your energy level? |
|  | 22.Do you feel fatigued? |
|  | 23.Do you feel dry mouth? |
|  | 24.Are you prone to catching colds? |
|  | 25.Do you have low-grade fever? |
|  | 26.Do you have lower back soreness? |
|  | 27.Are your bowel and bladder functions normal? |
|  | 28.Did you notice cyanosis (your lips, nails, skin, etc. turned purple?) |
| Psychological  domain | 29.Does coughing make you feel anxious? |
|  | 30.Does coughing make you feel annoyed or distressed? |
|  | 31.Does coughing make you feel depressed? |
|  | 32.Does coughing make you feel embarrassed? |
|  | 33.Does coughing make you feel frustrated or irritable? |
|  | 34.Does coughing make you feel scared or panicked? |
|  | 35.Do you have confidence in your treatment? |
|  | 36.Have you lost interest in things you used to enjoy? |
|  | 37.Are you worried about being discriminated against because of your condition? |
|  | 38.Are you worried that your condition will gradually worsen? |
|  | 39.Do you feel disappointed in the struggle with your illness? |
|  | 40.Do you feel worthless because of your condition? |
|  | 41.Are you worried that your health will deteriorate? |
| Environmental domain | 42.Does your condition worsen upon exposure to irritating odors (paint, cooking fumes, chemical fuels)? |
|  | 43.Does your condition worsen with seasonal changes? |
|  | 44.Is your condition affected by weather changes (overcast days, smog, wind, rain)? |
|  | 45.Is your condition affected by confined or poorly ventilated environments? |
| Social domain | 46.Does your condition affect your work? |
|  | 47.Does your condition affect your daily life? |
|  | 48.Does your condition trouble your family, friends, or neighbors? |
|  | 49.Does your condition affect your outdoor entertainment or leisure activities? |
|  | 50.Does your condition make you feel like a burden to others? |
|  | 51.Does your condition affect your social activities (visiting relatives/friends)? |
|  | 52.Does coughing interrupt your conversations or phone calls? |
|  | 53.Does treating your condition require family support (e.g., emotional encouragement, financial support, daily care)? |
| Satisfaction domain | 54.Do you think your current health status is good? |
|  | 55.Are you satisfied with the current treatment outcome? |
|  | 56.Are you satisfied with the improvement in your symptoms? |
|  | 57.Are you satisfied with your ability to perform daily activities? |
|  | 58.Are you satisfied with the care and support from others (family, friends, colleagues, neighbors, medical staff)? |
|  | 59.Do you have confidence in the current treatment methods? |
|  | 60.Are you worried about the side effects of medications? |

**Psychometric item reduction**

In the first field survey, 298 questionnaires were returned and 295 were analyzed. Thirty-three unique items were removed. Method-specific criteria flagged 10 items for limited variability, six for internal consistency, five for weak item–domain correlations, seven for low factor loadings, and 27 for item response theory parameters; these counts were not additive because some items failed multiple criteria. In the second survey, 225 questionnaires were returned and 223 were analyzed. Variability, internal consistency, correlation, and structural equation modelling criteria flagged six, six, two, and six items, respectively. Following expert adjudication, ten items were removed, the hemoptysis and common-cold items were retained because of their clinical relevance, and two overlapping items were merged. The remaining 16 items formed the final BE-PRO. Item-level results are reported in Tables S6 and S7, and the complete instrument is provided in Supplementary Instrument S1.

**Table S6. Item-level results from the first field survey**

| **Item** | **Discrete trend** | **Cronbach's α coefficient** | **Correlation coefficient** | **Factor analysis** | **IRT** | | | **Selected items** |
| --- | --- | --- | --- | --- | --- | --- | --- | --- |
|  |  |  |  |  | discrimination parameters | difficulty parameters | mean information |  |
| 1 |  |  |  |  |  |  |  | **√** |
| 2 |  | **×** | **×** |  |  | **×** | **×** | **×** |
| 3 | **×** | **×** | **×** |  |  | **×** | **×** | **×** |
| 4 |  | **×** | **×** |  |  | **×** | **×** | **×** |
| 5 |  | **×** | **×** |  |  | **×** | **×** | **×** |
| 6 |  |  |  |  |  |  |  | **√** |
| 7 |  | **×** | **×** |  |  | **×** | **×** | **×** |
| 8 |  |  |  |  |  | **×** | **×** | **×** |
| 9 |  |  |  |  |  |  |  | **√** |
| 10 |  |  |  |  |  |  |  | **√** |
| 11 |  | **×** |  |  |  | **×** | **×** | **×** |
| 12 |  |  |  |  |  |  |  | **√** |
| 13 |  |  |  |  |  |  |  | **√** |
| 14 |  |  |  |  | **×** |  |  | **×** |
| 15 |  |  |  |  | **×** |  |  | **×** |
| 16 |  |  |  |  | **×** |  |  | **×** |
| 17 |  |  |  |  | **×** |  |  | **×** |
| 18 |  |  |  |  |  |  |  | **√** |
| 19 |  |  |  |  |  |  |  | **√** |
| 20 |  |  |  |  |  |  |  | **√** |
| 21 |  |  |  |  |  |  |  | **√** |
| 22 | **×** |  |  |  | **×** |  |  | **×** |
| 23 | **×** |  |  |  | **×** |  |  | **×** |
| 24 |  |  |  |  |  |  |  | **√** |
| 25 | **×** |  |  |  | **×** |  |  | **×** |
| 26 | **×** |  |  |  | **×** |  |  | **×** |
| 27 |  |  |  |  | **×** |  |  | **×** |
| 28 |  |  |  |  |  |  |  | **√** |
| 29 |  |  |  |  |  |  |  | **√** |
| 30 |  |  |  |  | **×** |  |  | **×** |
| 31 |  |  |  |  | **×** |  |  | **×** |
| 32 |  |  |  |  | **×** |  |  | **×** |
| 33 |  |  |  |  | **×** |  |  | **×** |
| 34 |  |  |  |  |  |  |  | **√** |
| 35 |  |  |  |  |  |  |  | **√** |
| 36 |  |  |  | **×** |  |  |  | **×** |
| 37 |  |  |  | **×** |  |  |  | **×** |
| 38 |  |  |  |  |  |  |  | **√** |
| 39 |  |  |  | **×** |  |  |  | **×** |
| 40 |  |  |  | **×** |  |  |  | **×** |
| 41 |  |  |  | **×** |  |  |  | **×** |
| 42 |  |  |  |  |  |  |  | **√** |
| 43 |  |  |  |  |  |  |  | **√** |
| 44 |  |  |  |  |  |  |  | **√** |
| 45 |  |  |  |  |  |  |  | **√** |
| 46 |  |  |  |  |  |  |  | **√** |
| 47 |  |  |  |  |  |  |  | **√** |
| 48 |  |  |  |  | **×** |  |  | **×** |
| 49 | **×** |  |  |  | **×** |  |  | **×** |
| 50 |  |  |  |  |  |  |  | **√** |
| 51 | **×** |  |  |  | **×** |  |  | **×** |
| 52 | **×** |  |  |  | **×** |  |  | **×** |
| 53 | **×** |  |  |  | **×** |  |  | **×** |
| 54 |  |  |  |  |  |  |  | **√** |
| 55 |  |  |  |  |  |  |  | **√** |
| 56 |  |  |  | **×** |  |  |  | **×** |
| 57 |  |  |  |  |  |  |  | **√** |
| 58 |  |  |  |  | **×** |  |  | **×** |
| 59 |  |  |  |  |  |  |  | **√** |
| 60 | **×** |  |  | **×** | **×** |  |  | **×** |

×: Items recommended to be deleted; √: Items recommended to be retained.

**Table S7. Item-level results from the second field survey**

| **Item** | **Discrete trend** | **Cronbach's α coefficient** | **Correlation coefficient** | **Factor analysis** | **Structural equation modeling fit and evaluation** | **Selected items** |
| --- | --- | --- | --- | --- | --- | --- |
| 1 |  |  |  |  |  | **√** |
| 6 |  |  |  |  |  | **√** |
| 9 | **×** | **×** | **×** |  | **×** | **“√”** |
| 10 |  |  |  |  |  | **√** |
| 12 |  |  |  |  |  | **√** |
| 13 |  |  |  |  |  | **√** |
| 18 |  |  |  |  |  | **√** |
| 19 |  |  |  |  | **×** | **×** |
| 20 | **×** |  |  |  | **×** | **×** |
| 21 | **×** |  |  |  |  | **×** |
| 24 | **×** |  |  |  | **×** | **“√”** |
| 28 |  |  |  |  | **×** | **×** |
| 29 |  |  |  |  |  | **√** |
| 34 |  |  |  |  |  | **√** |
| 35 |  |  |  |  |  | **√** |
| 38 | **×** | **×** |  |  |  | **×** |
| 42 |  | **×** |  |  |  | **×** |
| 43 |  | **×** | **×** |  |  | **×** |
| 44 |  |  |  |  |  | **√** |
| 45 |  |  |  |  |  | **√** |
| 46 |  |  |  |  |  | **“×”** |
| 47 |  | **×** |  |  | **×** | **×** |
| 50 |  |  |  |  |  | **√** |
| 54 |  |  |  |  |  | **√** |
| 55 |  |  |  |  |  | **√** |
| 57 |  |  |  |  |  | **√** |
| 59 | **×** | **×** |  |  |  | **×** |

×: Items recommended to be deleted; √: Items recommended to be retained.

“√”: Items initially recommended for deletion by statistical analysis methods but retained after expert discussion**;** “×”: Items not recommended for deletion by statistical analysis methods but removed after expert discussion.

**Table S8. Independent-samples t-test results for the BE-PRO**

| **Item** | **t** | **Mean Difference** | **Std. Error Difference** | **95% Confidence Interval of the Difference** | | **P-values**  **two-sided** |
| --- | --- | --- | --- | --- | --- | --- |
|  |  |  |  | Lower | Upper |  |
| 1 | -9.823 | -1.475 | 0.150 | -1.774 | -1.176 | *P*<0.001 |
| 2 | -7.992 | -1.375 | 0.172 | -1.718 | -1.032 | *P*<0.001 |
| 3 | -7.904 | -1.300 | 0.164 | -1.627 | -0.973 | *P*<0.001 |
| 4 | -10.635 | -1.650 | 0.155 | -1.959 | -1.341 | *P*<0.001 |
| 5 | -12.576 | -1.875 | 0.149 | -2.172 | -1.578 | *P*<0.001 |
| 6 | -11.230 | -1.775 | 0.158 | -2.090 | -1.460 | *P*<0.001 |
| 7 | -10.388 | -1.625 | 0.156 | -1.936 | -1.314 | *P*<0.001 |
| 8 | -11.656 | -1.775 | 0.152 | -2.078 | -1.472 | *P*<0.001 |
| 9 | -10.145 | -1.475 | 0.145 | -1.764 | -1.186 | *P*<0.001 |
| 10 | -5.575 | -1.000 | 0.179 | -1.357 | -0.643 | *P*<0.001 |
| 11 | -6.599 | -1.100 | 0.167 | -1.432 | -0.768 | *P*<0.001 |
| 12 | -7.366 | -1.200 | 0.163 | -1.524 | -0.876 | *P*<0.001 |
| 13 | -13.549 | -1.875 | 0.138 | -2.151 | -1.599 | *P*<0.001 |
| 14 | -8.883 | -1.400 | 0.158 | -1.714 | -1.086 | *P*<0.001 |
| 15 | -11.814 | -1.450 | 0.123 | -1.694 | -1.206 | *P*<0.001 |
| 16 | -9.531 | -1.425 | 0.150 | -1.723 | -1.127 | *P*<0.001 |

**Table S9. Item discrimination and difficulty parameters for the BE-PRO**

| **Item** | **Discrimination parameter (a)** | **Difficulty parameter (b)** | | | |
| --- | --- | --- | --- | --- | --- |
|  |  | b1 | b2 | b3 | b4 |
| 1 | 1.85 | -2.42 | -0.99 | 0.20 | 3.09 |
| 2 | 1.62 | -2.33 | -1.19 | 0.23 | 3.37 |
| 3 | 1.46 | -2.71 | -1.09 | 0.75 | 3.30 |
| 4 | 1.52 | -1.62 | 0.09 | 1.46 | 6.88 |
| 5 | 1.54 | -1.56 | 0.08 | 1.08 | 3.96 |
| 6 | 2.27 | -1.68 | -0.53 | 0.59 | 3.15 |
| 7 | 2.17 | -1.48 | -0.21 | 1.10 | 3.21 |
| 8 | 2.18 | -1.51 | -0.12 | 0.97 | 6.20 |
| 9 | 1.99 | -1.35 | 0.17 | 1.59 | 6.30 |
| 10 | 0.84 | -4.55 | -2.15 | -0.23 | 4.41 |
| 11 | 0.93 | -4.41 | -1.65 | -0.09 | 4.06 |
| 12 | 0.81 | -3.89 | -0.87 | 1.45 | 6.63 |
| 13 | 2.41 | -1.69 | -0.45 | 0.64 | 2.74 |
| 14 | 2.13 | -1.53 | 0.00 | 1.43 | 5.62 |
| 15 | 2.70 | -2.65 | -0.61 | 0.82 | 2.92 |
| 16 | 1.97 | -2.39 | -0.25 | 1.02 | 2.71 |

**Final 16-item Patient-Reported Outcome scale for Bronchiectasis (BE-PRO)**

Please read each item and select the response that best describes your experience during the past 2 weeks. Select one response per item.

| **Example:** Did you have fatigue? | | | | |
| --- | --- | --- | --- | --- |
| Never  0 | Hardly ever  1 | Several times  2 | Many times  3 | Almost all the time  4 |

**√**

| 1. Do you have a cough? | | | | |
| --- | --- | --- | --- | --- |
| Never  0 | Hardly ever  1 | Several times  2 | Many times  3 | Almost all the time  4 |
| 2. Do you produce sputum? | | | | |
| Never  0 | Hardly ever  1 | Several times  2 | Many times  3 | Almost all the time  4 |
| 3. Do you have hemoptysis? | | | | |
| Never  0 | Hardly ever  1 | Several times  2 | Many times  3 | Almost all the time  4 |
| 4. Do you produce a large amount of sputum? | | | | |
| Not at all  0 | Very slight  1 | Moderate  2 | Serious  3 | Very serious  4 |
| 5. Do you have chest tightness? | | | | |
| Never  0 | Hardly ever  1 | Several times  2 | Many times  3 | Almost all the time  4 |
| 6. Do you have wheezing? | | | | |
| Never  0 | Hardly ever  1 | Several times  2 | Many times  3 | Almost all the time  4 |
| 7. Does your condition repeatedly progress or worsen? | | | | |
| Never  0 | Hardly ever  1 | Several times  2 | Many times  3 | Almost all the time  4 |
| 8. Are you prone to catching colds? | | | | |
| Never  0 | Hardly ever  1 | Several times  2 | Many times  3 | Almost all the time  4 |
| 9. Does coughing make you feel anxious, panicked, or fearful? | | | | |
| Never  0 | Hardly ever  1 | Several times  2 | Many times  3 | Almost all the time  4 |
| 10. Does coughing make you feel annoyed or even distressed? | | | | |
| Never  0 | Hardly ever  1 | Several times  2 | Many times  3 | Almost all the time  4 |
| 11. Does your condition worsen with seasonal changes? | | | | |
| Never  0 | Hardly ever  1 | Several times  2 | Many times  3 | Almost all the time  4 |
| 12. Is your condition affected by weather changes (e.g., overcast days, smog, wind, or rain)? | | | | |
| Never  0 | Hardly ever  1 | Several times  2 | Many times  3 | Almost all the time  4 |
| 13. Does your condition affect your daily life? | | | | |
| Never  0 | Hardly ever  1 | Several times  2 | Many times  3 | Almost all the time  4 |
| 14. Does your condition make you feel like a burden to others? | | | | |
| Never  0 | Hardly ever  1 | Several times  2 | Many times  3 | Almost all the time  4 |
| 15. Do you think your current health status is good? | | | | |
| Very poor  0 | Relatively poor 1 | Moderate  2 | Relatively good 3 | Very good  4 |
| 16. Are you satisfied with your ability to perform daily activities? | | | | |
| Very dissatisfied 0 | Relatively dissatisfied  1 | Moderate  2 | Relatively satisfied 3 | Very satisfied  4 |

**The end**

*Thank you for your participation!*

The BE-PRO comprises five domains: Physiology (items 1–8), Psychology (items 9–10), Environment (items 11–12), Society (items 13–14), and Treatment Satisfaction (items 15–16).

Scoring: The total score is the sum of all 16 item scores. Domain scores are the means of the corresponding item scores: Physiology, items 1–8; Psychology, items 9–10; Environment, items 11–12; Society, items 13–14; and Treatment Satisfaction, items 15–16.

The BE-PRO is copyrighted. It may not be altered, sold (paper or electronic), translated or adapted for another medium without the permission of Yang Xie, First Affiliated Hospital of Henan University of Chinese Medicine, No. 19 Renmin Road, Zhengzhou, Henan, 450046, People’s Republic of China.
